# Determination of Pollution and Environmental Risk Assessment of Stormwater and the Receiving River, Case Study of the Sudół River Catchment, Poland

**DOI:** 10.3390/ijerph20010504

**Published:** 2022-12-28

**Authors:** Izabela Godyń, Marek Bodziony, Agnieszka Grela, Krzysztof Muszyński, Justyna Pamuła

**Affiliations:** Faculty of Environmental Engineering and Energy, Politechnika Krakowska, 31-155 Kraków, Poland

**Keywords:** land use, stormwater quality, contamination, biogenic compounds, heavy metals, petroleum hydrocarbons, PAHs, toxicity potential, environmental risk assessment

## Abstract

Changes in the land use of urban catchments and the discharge of stormwater to rivers are causing surface water pollution. Measurements were taken of the quality of discharged stormwater from two areas with different types of development: a residential area and a residential–commercial area, as well as the quality of the Sudół River water below the sewer outlets. The following indicators were studied: TSS, COD, N–NO_3_, N–NO_2_, TKN, TN, TP, Zn, Cu, Hg, HOI, and PAHs. The influence of land use on the magnitudes of flows in the river was modeled using the SCS–CN method and the Snyder Unit Hydrograph Model. The results showed an increase in sealing and a resulting increase in surface runoff. Concentrations of pollutants in stormwater and analysis of the potential amounts of loadings contributed by the analyzed stormwater outlets indicate that they may be responsible for the failure to meet environmental targets in the Sudół River. Environmental risk assessment shows that the aquatic ecosystem is at risk. A risk factor indicating a high risk of adverse environmental effects was determined for N–NO_3_, Zn, and Cu, among others.

## 1. Introduction

Progressive urban development is adversely affecting the quality of surface water and the performance of sewage systems. This impact can be clearly seen through the disruption of the natural, dynamic, quantitative balance between precipitation and surface runoff processes. Urban catchments are characterized by a dynamic increase in sealed surfaces as a result of the construction of new buildings, roads, sidewalks, or parking lots, which contribute primarily to rapid and fast surface runoff [1], but also to an increase in the concentrations of pollutants entering the receiving watershed. The immediate cause is runoff from roofs, roads, or parking lots, which transport more and more pollutants in an increasingly shorter time [2]. Another factor that negatively affects surface water quality is the development of automobile transportation. It indirectly affects water quality because it is the source of a large amount of various pollutants entering the air.

As a result of the greenhouse effect, an increase in the number of extreme weather events, e.g., hurricanes, droughts, and heavy rainfall, is observed every year, thus determining the need to change the approach to the design of sewage systems [3]. There is a constant search for more and more effective tools for predicting possible hydrological risks and for the assessment of the functioning of the network (its overloading) and individual elements of the stormwater or combined sewer system [4,5].

Another important environmental issue, mainly for surface water quality reasons, is the development of methods for estimating the impact of rapid precipitation on the stormwater recipient, taking into account both the quantity of surface runoff and its quality [6]. 

The contact of precipitation with airborne pollutants causes it to be already initially polluted. Subsequently, pollution occurs as a result of contact with the catchment area and the formation of surface runoff, where along its path the concentration of pollutants gradually increases, culminating in the incorporation of the sewer system into the receiver [7]. The largest loads of pollutants enter the receiver from high-intensity and short-duration precipitation. Extended over time, low-intensity precipitation does not contribute significant flush loads of pollutants. The level of stormwater pollution is also influenced by the processes of accumulation and leaching of pollutants both in the catchment area and in the drainage system [8]. The rate of pollutant build-up and wash-off is very variable and depends to a large extent on the development and location of the catchment. Additionally, the variability of these processes results from the variability and intensity of precipitation during the year, which determine the load remaining after rainfall and the variability in the occurrence and length of precipitation-free periods [9,10]. Similar relationships also apply to the quality of surface water, which deteriorates in particular during floods (pluvial and fluvial floods) as a result of increased inflow of pollutants from drainage systems and polluted surface runoff, and also as a result of erosion processes [11,12].

Depending on the type of catchment development, surface runoff varies, both hydraulically (culmination time, volume of runoff) and qualitatively (concentration, pollutant load). Surface runoff is often classified in relation to the type of development [13,14,15].

It cannot be overlooked that the progressive pollution of the environment (in this case, surface water) affects not only nature, but also human health. It is estimated [16] that in the last 10 years as many pollutants have entered the Earth’s environment as in the previous 70 years. This is because the rate of spread of pollutants is increasing, which means that, for example, cancer in humans will occur more frequently. This fact is also confirmed by the World Health Organization’s announcement that as many as 75% of human diseases are due to poor environmental conditions [16].

It is therefore necessary to take appropriate measures to improve water and wastewater management in urban areas. This involves conducting studies or a creating a continuous monitoring system for the quantity and quality of stormwater discharged into surface waters [17]. 

Actions that should also be taken in the long term include the use of the potential of the blue–green infrastructure (BGI) and the management of water at the place of precipitation [18,19]. There are a number of combinations of gray and green infrastructure elements that make it possible to combine individual functions—e.g., retention and infiltration reservoirs that stop water runoff and allow it to slowly seep into the ground— with sedimentation ponds, plant passages, and other bioretention solutions, apart from retention, that participate in mechanical and biological water purification. Changes to the existing underground rainwater drainage systems require significant financial outlay. Their way of functioning requires improvement, so efforts should be made to relieve the network by building retention reservoirs, retention and infiltration reservoirs, or rainwater management at the site of precipitation. A number of studies prove that a greater degree of implementation of green solutions brings lower maintenance costs and better reduction of the amount of surface runoff and improvement in its quality [20,21,22,23]. 

In urbanized areas in particular, there are many conflicting economic, social, and economic interests related to water management. Urban rivers have long been used at suitable sites to discharge sewage and stormwater, leading to severe damage to aquatic ecosystems, often to the point that they no longer provide ecosystem services to society [24,25]. The current approach is primarily to pay attention to and properly value ecosystem services and to implement the idea of sustainable development [26,27,28]. Among the many benefits of this approach are those that relate to the environmental and social impacts. The environmental impact includes the following benefits: (a) reduction of rapid floods in watercourses, (b) reduction of water pollution, and (c) improvement of soil and water conditions in the catchment area. In turn, benefits of a social nature include: (a) eliminating (at least partially) losses due to flooding, (b) strengthening the ecological awareness of the inhabitants, and (c) improving the aesthetic values of urban areas [17].

Despite a number of studies cited above, it is still unclear what impact urban development without proper stormwater management has on surface water quality. Recognizing these problems, the present study investigated the quality of surface water runoff from urbanized areas and the quality of receiver waters. The aim of the study was to identify pollutant emissions from stormwater drainage systems in urbanized areas in the studied real catchment area of the Sudół River in Krakow. Additional objectives were: analysis of the impact of stormwater pollution on the quality of the Sudół River (threat to achieving the environmental goal: good water status) as well as assessment of environmental risk, i.e., the likelihood of negative effects as a result of exposure to potentially toxic environmental pollution. It is possible to assess the threat to achieving the environmental objective and the environmental risk in the studied catchment area using data from the measurement of concentrations of the following parameters: water quality in outflows from the stormwater drainage system and water quality of the Sudół River, to which stormwater is discharged. The influence of the type of development on the quality of surface runoff was evaluated by estimating and comparing the average concentrations for two different areas: (1) residential development and (2) commercial–service area. The results of pollutant concentrations determined in stormwater and the river were also compared to the limits contained in current regulations [29]. In addition, the authors compared changes in development in the study catchment in 2000 and 2018 and estimated how these changes affected the increase in surface runoff. The impact of the quality of discharged stormwater on the quality of the receiving water body—the Sudół River—was assessed. An innovative aspect of the work is the carrying out of an environmental risk assessment of pollution indicators for surface runoff from residential and service–communication areas and surface water to prevent environmental risks. 

## 2. Materials and Methods

### 2.1. Sudół River Catchment

**Figure 1:**
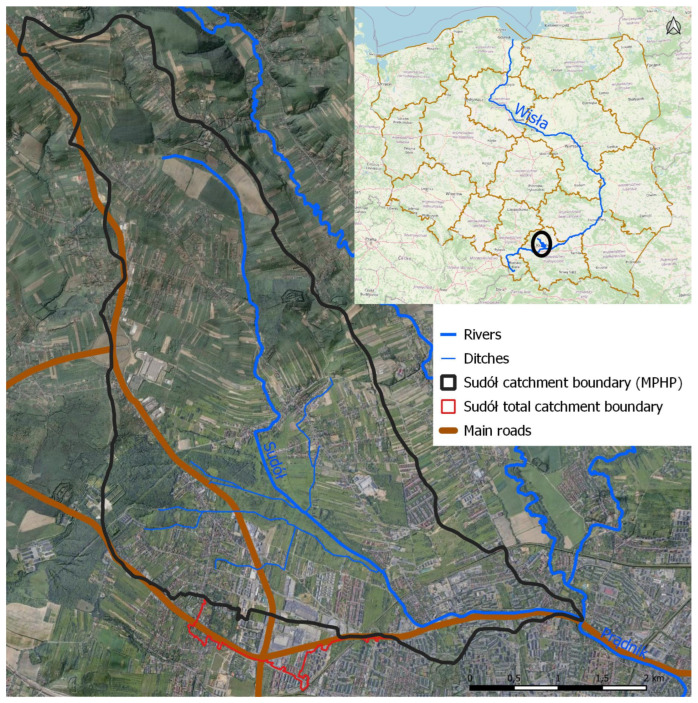
Location of the study area of the Sudół catchment with depiction of current land use based on an orthophotomap.

The studied catchment area of the Sudół River is located in the northern part of the city of Krakow and in the areas of the communes of Zielonki and Wielka Wieś. The 8.98-km-long Sudół is a right-bank tributary of the Prądnik River; its catchment area is 18.38 km^2^. The catchment area has a suburban character with a fairly diverse land use, predominantly agricultural and urbanized. In its southern part, within the administrative boundaries of the city of Krakow, industrial or commercial areas and low-density urban buildings predominate. In its central part, there are mainly areas of low-density housing, deciduous forests, meadows, and pastures, while the northern area is largely used for agriculture where there is low-density housing. Figure 1 shows the location of the study area against the background of an orthophotomap showing current land use with the main road transportation routes marked.

The boundary of the Sudół River catchment marked in Figure 1 does not entirely coincide with the boundary of the watershed according to the current Map of the Hydrological Division of Poland (MPHP). The reason for this is the heavily modified and anthropogenically transformed terrain equipped with a developed sewage infrastructure that also drains stormwater from neighboring catchments. Using GIS and SWAT software (QGIS 3.16: open-source, https://www.qgis.org (accessed on 20 November 2022); SWAT: USDA Agricultural Research Service, Temple, Texas, USA), the catchment area was verified by analyzing available databases, i.e., the Numerical Terrain Model (NMT), the National Integration of Utilities (KIUT), and base maps [30]. The result is an expansion of the catchment area of the Sudół River by an area of about 74.9 hectares served by stormwater drainage systems (area boundary marked in red in Figure 1). The drained areas are mainly road traffic routes; areas of industry, large-format trade, and services; and sealed areas of low- and high-intensity residential neighborhoods. 

Development of the catchment area of the Sudół River (including the sewer catchment area) includes, according to the Corine Land Cover database (CLC 2018) [31]:

- agriculture, arable land, cropping systems: 835.6 ha, 46.5% of the catchment area;
- residential development and sports areas: 530.6 ha, 29.5%;
- meadows, areas of grassy vegetation: 190.3 ha, 10.5%;
- forests, wooded areas: 139.3 ha, 8%;
- industrial and commercial areas: 100.2 ha, 5.5%.

Attention should also be paid to the major transportation routes highlighted in Figure 1, which, according to the Database of Topographical Objects (BDOT10k) [30], account for almost 2% of the catchment area (40.9 hectares). The Sudół catchment area includes the intersection of two national roads, some of the most important in the region, with a daily capacity of more than 45,000 vehicles per day [32], which can lead to increased pollution of air and rainwater with particulate matter, heavy metals, petroleum substances, and polycyclic aromatic hydrocarbons (PAHs).

The Sudół River belongs to the Prądnik surface water body (JCWP Prądnik RW200006213749), which is characterized as an upland watercourse on carbonate substrate (type RW_wap). According to current legislation [29], the environmental objectives for the studied watercourse, i.e., the limit values of surface water quality indicators for good status (Class II), are presented in Table 1.

The catchment area of the Sudół River is not monitored, but the higher-order river Prądnik is studied as part of the State Environmental Monitoring (PMŚ). The Sudół is the second largest of the Prądnik’s seven tributaries; the Sudół catchment constitutes about 10% of the Prądnik total catchment area (169.5 km^2^). The Prądnik is monitored at the estuarine measurement and control cross-section “Prądnik-Białucha Krakow Ujście” located about 4.7 km below the mouth of the Sudół River. The latest survey results (2020) show that the waters of the Prądnik reach [33]:

- Class III in terms of biological elements,
- Class II exceedances for physical and chemical elements, including class II exceedances for nutrients (TKN, N–NO_3_, N–NO_2_, TN, P–PO_4_, and TP), and class II for particularly harmful substances (including class II concentrations of Zn, Cu, HOI).

### 2.2. Water Sampling and Research on the Quality of Surface Runoff and Waters of the Sudół River

#### 2.2.1. Sampling Locations

In order to identify the actual water quality status of the Sudół River and the impact of surface runoff from the stormwater drainage system on the Sudół water quality, the research work included measurement campaigns of river water quality and selected outflows from the stormwater drainage system.

**Figure 2:**
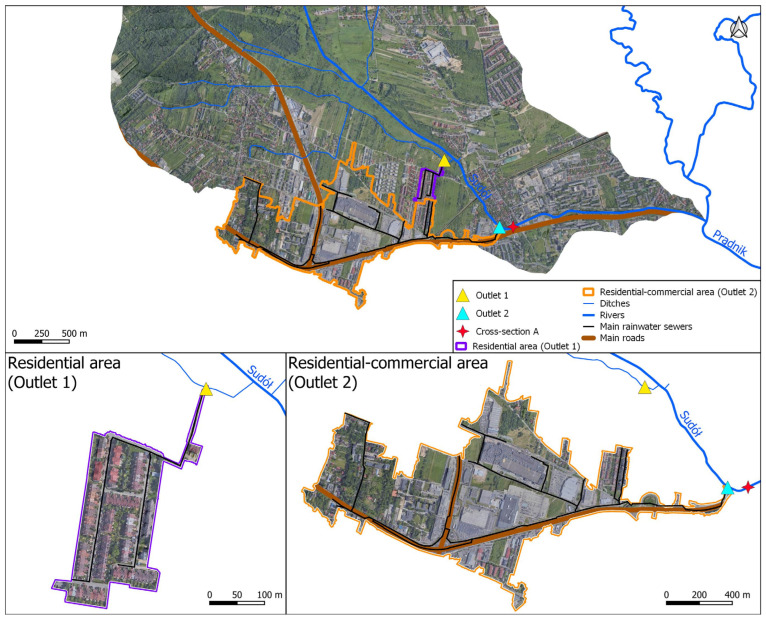
Catchment development in water sampling areas.

The study of surface runoff quality included two areas drained by stormwater drainage systems with discharge of stormwater to surface waters:

- residential area: an area of low-density urban development with about 118 terraced houses with home gardens; stormwater drainage also drains local access roads and pedestrian routes; stormwater drainage catchment area of 4.22 hectares; the length of the drainage network of 1.3 km with drainage of stormwater into the drainage ditch draining into the Sudół in 6.25 km, marked as outlet 1 in Figure 2,
- an area of industrial, commercial and communications land and low-density residential development: about 65 ha of single- and multi-family residential development; more than 54 ha of industrial and commercial land and roads; less than 1.3 ha of biologically active land (meadows and pastures), accounting for about 1% of the catchment area; a stormwater drainage system of about 53.4 km serves as a catchment area of 120.31 ha in total and discharges stormwater directly into the Sudół River in 7.01 km, marked as outlet 2 in Figure 2.

Waters of the Sudół River were sampled at 7.14 km (cross-section A in Figure 2), located about 130 m downstream of outlet 2, 1.83 km to the Sudół confluence with the Prądnik River. Cross-section A encloses a catchment area of about 17.2 km^2^.

#### 2.2.2. Stormwater and Surface Water Pollution Indicators Tested

The pollutants that occur in stormwater can be classified into several main categories: solids (insoluble), heavy metals, trace substances, organic compounds, herbicides, and petroleum substances [34,35]. Another classification is presented in the work [36], where 25 priority pollutants, i.e., the most dangerous for the environment and humans, were identified, which require special attention when studying the quality of stormwater. These pollutants were divided into five categories (Table 2).

For the analysis of surface runoff quality, 10 measurement cycles were carried out from 2019 to 2022 by sampling stormwater flowing from outlets 1 and 2 of the stormwater drainage systems indicated in Figure 2. 

Sudół waters were sampled and analyzed in three measurement cycles in 2022.

The study focused on selected groups of indicators characterizing: (a) physical condition: total suspended solids (TSS) concentration; (b) oxygen conditions and organic pollutants: chemical oxygen demand COD; (c) biogenic conditions: Kjeldahl nitrogen TKN, nitrate nitrogen N–NO_3_, nitrite nitrogen N–NO_2_, total nitrogen TN, and total phosphorus TP, were examined. In addition, specific synthetic and non-synthetic pollutants in the form of petroleum hydrocarbons (hydrocarbon oil index HOI) [37], as well as heavy metals such as zinc (Zn), copper (Cu), and mercury (Hg) were also evaluated. The assessment also included the determination of substances particularly harmful to the aquatic environment identified as water policy priority substances, which include polycyclic aromatic hydrocarbons (PAHs). The resulting PAH concentration is the sum of the concentrations of the compounds: benzo(b)fluoranthene, benzo(k)fluoranthene, benzo(a)pyrene, benzo(ghi)perylene and indeno(123-cd)pyrene.

Total suspended solids are considered the most significant water pollutant in urban areas [38]. Total suspended solids originate from dust fallout mainly from coal combustion, dust emissions, and pollutants emitted by transportation [39,40,41]. Total suspended solids have the ability to sorb other pollutants on their surface, such as heavy metals, petroleum substances, hydrocarbons, or nitrogen and phosphorus compounds, which is directly reflected in the results of other indicators tested, which are significantly exceeded. Increased suspended solids in the outlets from the drainage system may also be caused by leaching of sediments accumulated in collectors and rainwater wells. Suspended sediment load is related to flow, catchment parameters, and seasonality (season of the year or even month) [42,43].

Chemical oxygen demand (COD) was determined in waters because it conventionally determines the amount of chemically degradable substances. According to many studies, it showed strong correlations with the concentration of total suspended solids, among others [44]. Biogenic compounds are parameters that should be studied especially when green areas have a large share of the total catchment area or when the catchment includes agricultural land. Biogenic compounds include nitrogen and phosphorus, which are responsible for eutrophication, that is, excessive growth of periphyton (diatoms, green algae, and cyanobacteria) and macrophytes (algae, bryophytes). The most commonly studied are nitrate nitrogen, total nitrogen, and total phosphorus. The various forms of nitrogen in waters come from: atmospheric diffusion, surface runoff, domestic sewage, and industry. Sources of phosphorus in water can be rock weathering, soil erosion, leaching of phosphate minerals from the ground, and decomposition of plant and animal matter.

Heavy metals, which are a group of pollutants dangerous to both flora and fauna, are also determined in waters. Heavy metals are a byproduct of fuel combustion processes [45]. In addition, petroleum hydrocarbons (HOI) are determined in stormwater and river waters, the presence of which in water is due to human activities, which include the use of diesel, lubricating oils, and transformer oils in all machinery and vehicles [46]. Polycyclic aromatic hydrocarbons (PAHs) are also determined in water. Their presence in water can result from human activities, which include the burning of fossil fuels (under Polish conditions, the cause of their occurrence can be individual, distributed heating systems) and the burning of liquid fuels in vehicles with internal combustion engines as well as tire abrasion. PAHs emitted into the atmosphere can be freely transported over long distances and can cause water pollution [47].

#### 2.2.3. Methods for Determining Indicator Concentrations

The collected water samples from the drainage outlets and the Sudoł River were subjected to physical and chemical analysis at an accredited testing laboratory belonging to the Waterworks of the City of Krakow S.A. This laboratory holds a certificate number AB 776 issued by the Polish Accreditation Center [48]. Determinations of individual indicators were carried out using the following methods:

- total suspended solids by weight according to PN-EN 872:2007 + Ap1:2007;
- chemical oxygen demand (COD) by spectrophotometric method according to PN-ISO 15705:2005;
- Kjeldahl nitrogen concentration by spectrophotometric method according to PN-EN 25663: 2001;
- nitrate nitrogen concentration by spectrophotometric method according to PN-82/C-04576/08;
- nitrite nitrogen concentration by spectrophotometric method according to PN-EN 26777: 1999;
- total nitrogen concentration from calculations according to PN-73C-04576/14;
- total phosphorus concentration by spectrophotometric method according to PN-EN ISO 6878:2006 (pkt 7) + Ap1:2010 + Ap2:2010;
- hydrocarbon oil index (petroleum hydrocarbons) by gas chromatography with flame-ionization detection (GC–FID) according to PN-EN ISO 9377-2:2003;
- concentration of metals: zinc and copper by flame atomic absorption spectrometry (FAAS) according to PN-ISO 8288:2002, method A; mercury by atomic absorption spectrometry with amalgamation technique according to PB-W-38 issue 3, dated 04.01.2021.
- concentration of polycyclic aromatic hydrocarbons (PAHs) calculated as the sum of benzo(b)fluoranthene, benzo(k)fluoranthene, benzo(a)pyrene, ben-zo(ghi)perylene, and indeno(123-cd)pyrene determined by high performance liquid chromatography with fluorescence detection (HPLC–FLD) according to PN-EN ISO 17993:2005.

### 2.3. Surface Runoff Modeling

The change in development in urbanized areas causes an increase in soil sealing; the construction of buildings (residential, commercial, industrial, etc.) and accompanying infrastructure (roads, parking lots, pedestrian routes) creates impervious surfaces that hinder or prevent the infiltration of rainwater into the ground. Sealing of areas brings an increase in the share of surface runoff in the water balance, as well as increased inflow of pollutants. 

The average amount of annual precipitation in Krakow in the last multi-year period 1991–2021 was 673 mm. The average number of days with precipitation above 0.1 mm was 172 days, with the majority being precipitation with daily totals up to 10 mm (0.1–1 mm 65 days, 1–5 mm 66 days, 5–10 mm 24 days); precipitation with daily totals above 10 mm occurred on average for 17 days per year [49]. For the purpose of this study, a design precipitation of 19.38 mm (*p* = 20%, frequency of 1 in 5 years) determined from a local precipitation model for the city of Krakow was used.

#### 2.3.1. Precipitation Modeling

**Figure 3:**
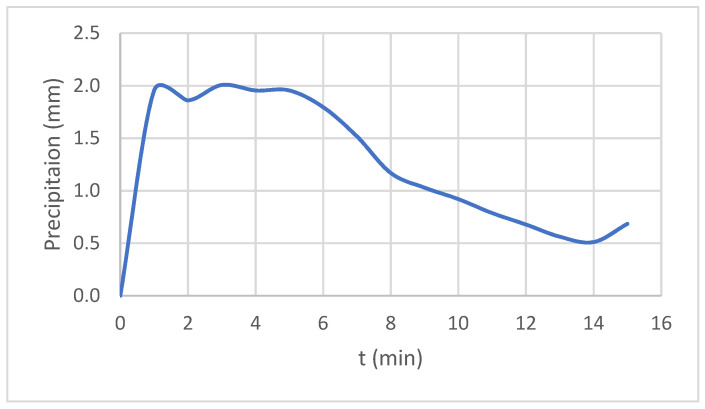
The design rainfall hyetograph type 1, precipitation with a probability of occurrence *p* = 20%.

For the modeling and sizing of the drainage system of the city of Krakow, the local precipitation model developed by Krakow Water [50] was used. The model was developed based on the distribution series of phase precipitation maxima for durations (phases) ranging from 5 to 4320 min, from observations in the period 1986–2019. Four local model hyetographs were also developed based on them. For the purposes of this work, in accordance with the recommendations of Krakow Water [50] and the Polish standard [51], rainfall with a probability of occurrence of *p* = 20% (frequency of 1 in 5 years) and a duration of 15 min was adopted for the design of infrastructure in urban centers, service areas, and industry for the design of drainage systems. The precipitation with such parameters is 19.38 mm, and its intensity is equal to 215.33 dm^3^/(s∙ha). The distribution of precipitation according to the type 1 hyetograph was adopted; the frequency of rainfall events with a distribution over time consistent with this hyetograph is one rainfall event per approximately three torrential rainfall events [50] (Figure 3).

#### 2.3.2. Runoff Modelling

A review of approaches to modeling the impact of sealing on the hydrology of urbanized catchments was done by Jacobson [52] and Lisennbee et al. [53] in review papers; other review papers include [54,55,56,57]. The aforementioned papers analyze ways to identify and quantify this impact, showing the most commonly used ways to model the impact of sealing include hydrological models such as HydroCAD, L–THIA, MIKE Products, MOUSE, MUSIC, SWAT, SWMM, and others. Additionally, modeling the surface runoff by applying the Soil Conservation Service Curve Number method (SCS–CN) is used [58,59,60,61,62,63].

In this study, hydrological modeling is conducted using:

- calculations of effective precipitation by the SCS–CN method,
- transformation of effective precipitation into surface runoff using the Snyder Unit Hydrograph Model.

#### 2.3.3. SCS–CN Method 

The SCS method is commonly used for hydrological modeling in both controlled and uncontrolled catchments [64,65]. This method to assess the impact of urbanization on changes in hydrology was used, among others, by Li et al. [66], who studied the hydrologic effects of urbanization on direct runoff characteristics in Shenyang (China) and by Sjöman and Gill [67] in the analysis of sealing due to changes in land use in the Höjeå river catchment (Sweden). In the SCS–CN method, effective precipitation depends on the soil group and land use of the catchment area. These factors are captured by the dimensionless parameter CN, taking values in the range of (0, 100]. The amount of excess rainfall is calculated from the formula [68,69]:

(1) $$P_{e}=\{\begin{array}{c} \frac{{\left(P-0.2S\right)}^{2}}{P+0.8S},\;when\;P\geq 0.2S\\ 0,\;when\;P<0.2S \end{array},$$

where:

$P_{e}$ = excess rainfall (mm),

P = total rainfall (mm),

S = maximum potential catchment retention (mm).

Maximum potential catchment retention *S* is determined by the relationship [68]:

(2) $$S=25.4\;\left(\frac{1000}{\mathrm{CN}}-10\right),$$

where:

CN is the parameter (curve number) of distribution of precipitation to runoff (0 = no runoff and 100 = total runoff).

In urban areas, the CN parameter—the curve number for a highly sealed surface, such as asphalt surfaces, from which almost all of the precipitation goes into surface runoff—is 98. For biologically active green surfaces such as lawns or urban parks, the curve number (CN) varies from 39 to 89, depending on the quality of the green areas and also on soil conditions (i.e., lower CN values for more permeable soils such as sands and high CN values for soils with high clay content) [67,69]. The SCS–CN method categorizes soils into one of four different groups: (A) sand, loamy sand, (B) silt loam or loam, (C) sandy clay loam, and (D) clay loam, silty clay loam, sandy clay, silty clay, or clay [67]. Table 3 summarizes the values of the CN parameter for the urban catchment area. 

For areas with variable development, the CN value can be determined as a weighted average from the formula:

(3) $$\mathrm{CN}={\mathrm{CN}}_{av.}=\frac{1}{A}\sum_{i=1}^{n}\left({\mathrm{CN}}_{i}\cdot A_{i}\right),$$

where: 

${\mathrm{CN}}_{av.}$ = average value of the parameter CN, 

${\mathrm{CN}}_{i}A_{i}$ = the value of the CN parameter for the *i*-th homogeneous surface, 

$A_{i}$ = area of the *i*-th homogeneous surface (km^2^), 

A = catchment area (km^2^).

#### 2.3.4. Snyder Unit Hydrograph Model

Quantitative modeling of runoff generation and transfer to the outlet is necessary to assess the impact of urbanization and land use on changes in hydrology. For ungauged catchments, modeling can be used by employing unit hydrograph models. To calculate the transformation of effective precipitation into surface runoff for the Sudół River, the HEC–HMS program was used, in which the Snyder Unit Hydrograph Model (SUHM) [70] is applied to determine the flow in a controlled catchment. 

The results of many studies indicate the accuracy and usefulness of the SUHM for derivation of the runoff hydrograph: Jena and Tiwari [71] applied SHUM in watersheds in the West Bengal state (India), and El Hassan et al. [72], Haibo et al. [73], and Babu et al. [74] used the SUHM (embedded in the HEC–HMS) to simulate rainfall-runoff processes. Other applications concerned the development of a synthetic unit hydrograph, e.g., for arid catchments (Oman) [75] and applications of SUHM to assess flood hazards in Georgia [76] and in India [77,78]. A review of other SUHM applications is also presented by Bahrami et al. [79]. Polish research includes work by Młyński et al. [68] that analyzes the possibility of using selected rainfall-runoff models, including the Snyder UHM, to determine the design hydrograph and the associated peak flow in a mountain catchment, showing fewer errors of the EBA4SUB model than the Snyder UH and NRCS–UH models and pointing to it as an alternative to these models.

SHUM is based on the concept of the unit hydrograph, whose basic parameters are: the lag $t_{p}$, peak flow $U_{p}$, and total time base $t_{r}$ [70]. The parameters of the mathematical model are estimated based on certain physiographic characteristics of the catchment (for uncontrolled catchments) or can be determined by optimization methods (for controlled catchments). 

The time of occurrence of the culmination of the unit hydrograph $t_{p}$ is calculated from the formula [70]:

(4) $$t_{p}=5.5\cdot t_{r},$$

where:

$t_{p}$ = the basin lag (h),

$t_{r}$ = standard duration of effective rainfall (h).

Using the catchment parameters, the lag time can be determined from the equation [70]:

(5) $$t_{p}=0.75\cdot C_{t}\cdot {\left(L\cdot L_{C}\right)}^{0.3},$$

where: 

$C_{t}$ = basin coefficient related to catchment area retention ($C_{t}$ = 1.8–2.2),

L = length of the main stream from the outlet to the divide (km),

$L_{C}$ = length along the main stream from the outlet to a point nearest the watershed centroid (km).

The culminating flow $U_{p}$ is calculated from the formula [70]:

(6) $$\frac{U_{p}}{A}=2.75\frac{C_{p}}{t_{p}},$$

where: 

$C_{p}$ = Snyder model parameter related to catchment retention ($C_{p}$ = 0.4–0.8);

*A* = catchment area (km^2^).

*t_p_* = the basin lag (h).

**Figure 4:**
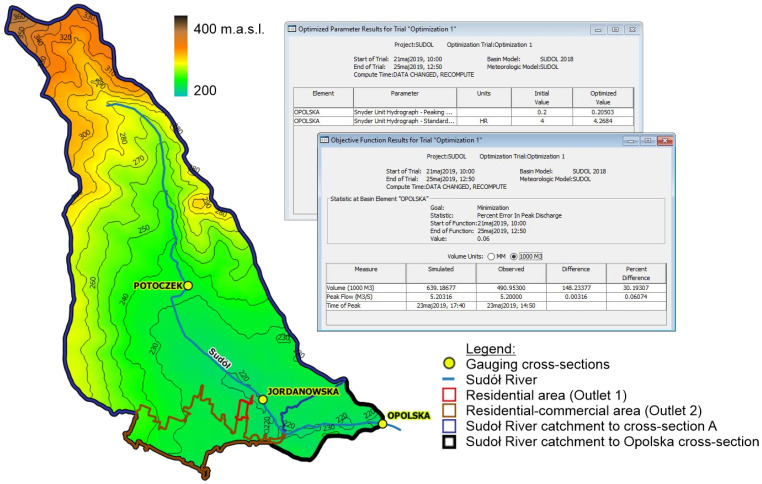
Structure and tabular summary of the results of the calibration of the mathematical model of the catchment area of the Sudół River.

Within the framework of the present study, a mathematical model of the catchment area of the Sudół River was developed based on data from the automatic measurement system established in the Sudół River catchment [80]. Location of three gauging cross-sections (Potoczek, Jordanowska, and Opolska) of the measurement system on the Sudół River is presented in Figure 4.

The model was run using the HEC–HMS 4.7 computer program. 

Two basic components of a streamflow hydrograph are: (1) direct runoff and (2) baseflow [70,81]. Baseflow is the sustained or “fair-weather” runoff of prior precipitation that was stored temporarily in the watershed, plus the delayed subsurface runoff from the current storm. 

There are three methods of separation of baseflow: straight line, fixed base, and variable gradient [70,81]. The Sudoł model assumed a constant, monthly-varying value of the baseline flow (a standard option of the HEC–MS model), which was determined on the basis of data from the automatic measurement system of the Sudół River. For the month of May 2019, the base flow was 0.07 m^3^/s.

The topography of the catchment area and the hydrographic network is shown in Figure 4.

**Figure 5:**
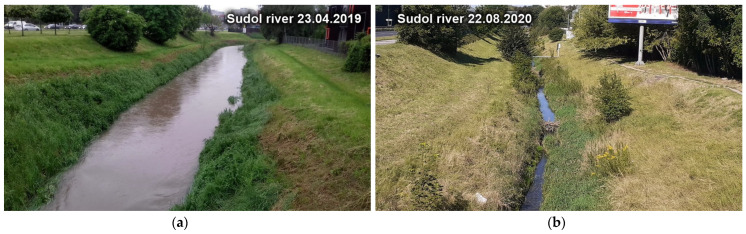
The water level in the Sudół River: (**a**) April 2019 (high water); (**b**) August 2020 (low water).

Since the maximum flow was the most important variable in the calculations, the rainfall of May 2019, which caused high levels and flows in the Sudół watercourse, was adopted for the calibration of the model. Figure 5 shows the water level of April 2019 (high water) and August 2020 (low water).

**Figure 6:**
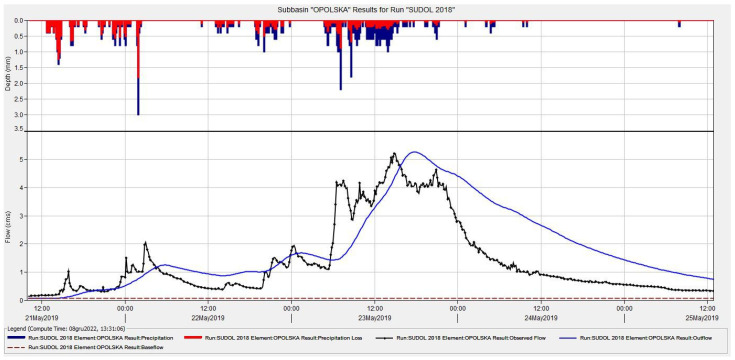
Graphical summary of model calibration results for the Sudół River catchment area.

The model calibration used precipitation-flow data from 2019 for the Opolska gauging cross-section (Figure 6). For calculation purposes, each area was defined as an independent catchment. The model used two methods:

- SCS to calculate the effective precipitation based on the CN parameter, according to Equations (1)–(3),
- SNYDER UHM (Standard) to calculate the transformation of effective precipitation into surface runoff for a controlled catchment according to Equations (4)–(6).

The following parameters of the Snyder model were assumed for the calculations:

- Standard Lag (HR): 4.2684
- Peaking Coefficient: 0.20503.

### 2.4. Water Quality and Enviromental Risk Assesment

#### 2.4.1. Estimation of Pollutant Load

For the studied pollutant indicators, the value of the load flowing with runoff from the catchment was determined using the formula [82]:

(7) PL=0.0864·PC·Q ,

where:

PL = pollutant load (g/s),

PC = pollutant concentration (mg/L),

Q = outflow from the sewer outlet/flow in the river (m^3^/s).

#### 2.4.2. Environmental Risk Assessment

Based on the risk quotient (RQ), the potential ecological risk of organic compounds on aquatic ecosystems is assessed, that is, the likelihood of negative effects due to exposure to potentially toxic environmental contaminants [83]. RQ can be calculated for three representative trophic levels of an aquatic ecosystem, which are fish, invertebrates, and algae [84]. Risk analysis involves comparing the maximum measured environmental concentration of a given compound, i.e., measured environmental concentration (MEC), to the predicted concentration of the substance below which no harmful environmental effects are observed, i.e., predicted no effect concentration (PNEC) [85]. RQ values for individual indicators were calculated according to the formula [86,87]:

(8) $$\mathrm{RQ}=\frac{\mathrm{MEC}}{\mathrm{PNEC}},$$

To determine the environmental risk for each indicator, the following classification was adopted [88]:

RQ < 1 = no environmental risk,

1 ≤ RQ < 10 = there is little potential for adverse effects, 

10 ≤ RQ < 100 = potential for adverse effects is significant,

RQ ≥ 100 = adverse effects are to be expected.

PNEC values were taken from the available literature (Table 4). If more than one value was found for the analyzed compound for a given trophic level, the lowest value was taken into account, thus reflecting the environmental risk for the most sensitive species [89]. The PNEC is determined as a quotient of the results of toxicity tests, consisting of the determination of the concentration of a substance causing specific harmful effects and the corresponding value of a safety factor selected according to the guidelines of Directive 2000/60/EC [90,91].

## 3. Results

### 3.1. Changes in Land Use in the Sudół Catchment Area

**Figure 7:**
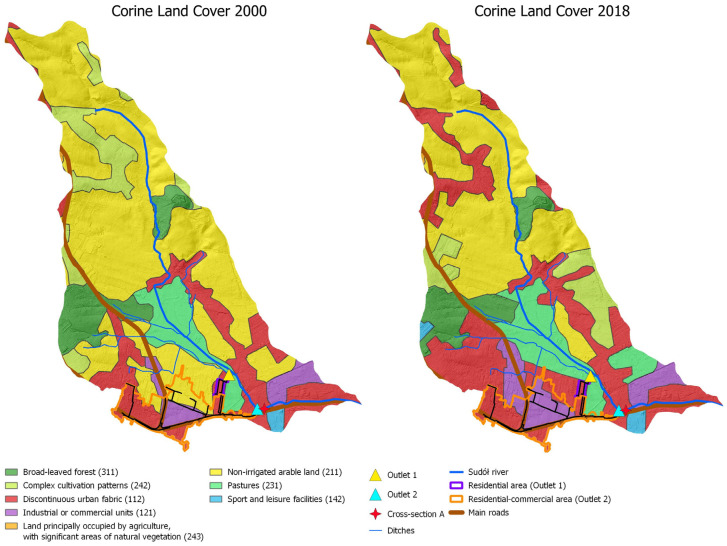
Illustration of changes in the development of the studied catchment of the Sudół River using CLC 2000 and CLC 2018 data.

Analysis of the collected data allowed us to characterize the study area, taking into account the change in land use according to Corine Land Cover from 2000 and 2018. The result of the study is illustrated below in Figure 7; the changes in the development of the study area took place over 18 years. The progressive urbanization of urban and suburban space is clearly visible. 

**Figure 8:**
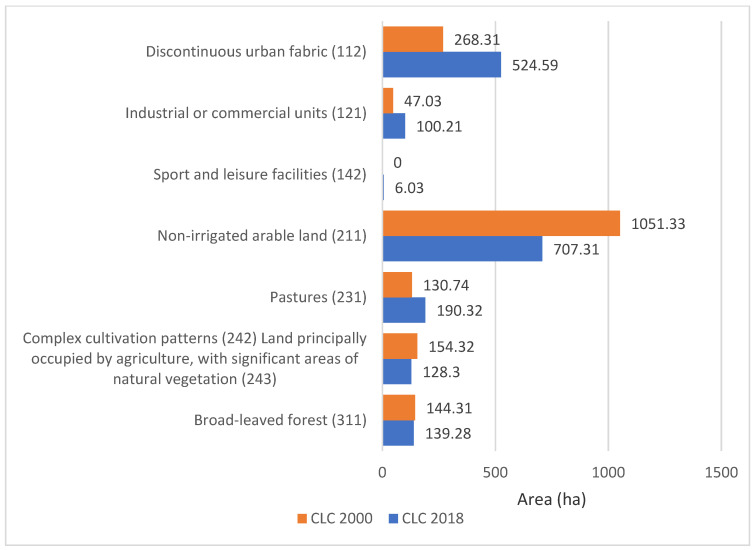
Summary of changes in land use of the total catchment area of the Sudół River in 2000 and 2018, according to Corine Land Cover.

The analysis shows that in the process of urbanization, built-up areas are replacing biologically active areas. The consequence is a systematic increase in sealed areas. In the catchment area of the Sudół River, a high increase in industrial and commercial areas (an increase of 113%) and residential areas (by more than 96%) is evident. A summary of the various forms of land use in 2000 and 2018 is provided in Figure 8.

Figure 8 also shows that during the analyzed time period of 2000–2018, in addition to a noticeable decrease in biologically active land (arable land, deciduous forests), there was also a favorable increase in land covered with disorganized greenery in the form of meadows and pastures. This is estimated to be an increase of about 60 hectares (46%). The area of forests, although it decreased by 3.5%, still represents nearly 140 hectares of significant area located in the upper sections of the ditches supplying water to the Sudół River. Thus, it continues to play a retention role, reducing or at least delaying the outflow of stormwater through the ditches. 

Development in the two urbanized areas analyzed, from which stormwater is discharged by the stormwater drainage system to the Sudół River, also underwent changes in the period 2000–2018:

- The residential area with stormwater drainage through outlet 1: this area has not been subject to significant changes in development during the period under review; vegetation areas slightly reduced their area by 0.01 hectares in favor of residential development.
- Residential and commercial area with stormwater drainage through outlet 2: changes are definitely more pronounced; the area was subject to intensive development in the period 2000–2018. There has been a liquidation of biologically active areas; all of the arable land present in 2000 has been replaced by sealed areas under industrial and commercial areas, an increase of 25.9 hectares or nearly 92%, and under low-density housing, an increase of 4.92 hectares or more than 8%. The calculations show that 31.76 hectares of land changed designation.

Using CLC 2018 coverage, development data, and soil data [98], the CN parameter was calculated (Table 5) together with the catchment area to perform calculations of rainfall transformation into surface runoff. The calculations used the precipitation scenario presented in Section 2.3.1. Calculations were carried out for two periods consistent with CLC 2000 and CLC 2018 data. 

### 3.2. Changes in Flow Due to Changes in Land Use

Table 6 summarizes the results of precipitation–flow transformation calculations for the precipitation scenario for the computational cross sections in the Sudół River drainage basin. Compiling the results of the calculations, it can be seen that in the period 2000–2018 there was an increase in flow (both maximum flow and flow volume). On the scale of the entire catchment, this is a change of 2.6%, which indicates increased sealing of the catchment area. There is a slight increase in flow in the catchment of outlet 1 (a residential area), where changes in development were small. The highest changes of more than 16% were recorded in the catchment of outlet 2 (residential–commercial area), where developed areas (residential neighborhoods and industrial and commercial areas) increased significantly.

### 3.3. Water Quality of the Sudół River

Three measurement cycles of water quality of the Sudół River were carried out in spring (sample taken in March), summer (sample taken in May), and autumn (September). Table 7 summarizes the results of measurements of concentrations of selected pollutants and also lists the minimum, average, and maximum concentrations.

Of the indicators tested, according to current legislation [29], limits have been set only for some of the indicators shown in Table 1. The remaining indicators have been tested (TSS, COD, TKN, and N–NO_2_), but according to current legislation, no accurate interpretation can be made due to the lack of limits to which the marked values can be compared. For these four indicators, the authors only made a general interpretation.

The concentration of suspended TSS reached its highest value of 180 mg/L in a sample taken in March, and it was more than 3 times higher than the concentration measured in September. In samples in which high concentrations of TSS were found, they were also accompanied by high concentrations of COD.

Among the biogenic compounds tested, the limits for Class II are set for total nitrogen, nitrate nitrogen, and total phosphorus (Table 1). For total nitrogen, the exceedance is twofold and for total phosphorus nearly fivefold. Biogenic compound pollution of the Sudół waters may also be the cause of Class II exceedances on the higher-order river, the Prądnik River, which are recorded in the monitoring [33]. 

The tested concentration of petroleum hydrocarbons HOI exceeded the permissible limit for Class II equal to 0.2 mg/L only in the sample from March 2022, and in the remaining samples the concentrations were below the minimum quantification level. Zn concentrations exceeded the permissible limits for Class II (Table 1). HOI and Zn contamination of Sudół waters, as in the case of biogenic compounds, can also be the cause of Class II exceedances on the upstream river Prądnik [33]. The study of Hg and Cu concentrations does not provide clear answers, as their concentrations were below the minimum quantification level.

PAHs are also determined in the waters. The results show that the limit comparable to benzo(a)pyrene was exceeded (Table 1). Of the three samples tested, the highest concentration of PAHs was recorded in a water sample from March 2022, when it also exceeded the permissible maximum concentration of PAHs.

### 3.4. Quality of Stormwater

There were 10 cycles of measurements of the quality of stormwater from 2 outlets of drainage systems into the Sudół River. Table 8 summarizes the results of pollutant concentrations measured in the outflow from the stormwater drainage system of a residential area, while Table 9 shows the results of measurements in the outflow from the stormwater drainage system covering a residential–commercial area.

The obtained values of concentrations were related to the limits for surface water presented in Table 1. The performed measurements allow for comparing the quality of stormwater discharged from areas with different intensities of development: (1) a low-intensity residential area with a local network of access roads, outlet 1, and (2) a residential–commercial area with a developed transportation network with heavy traffic, outlet 2:

- Average concentrations of biogenic compounds: the concentration of N–NO_3_ does not exceed standards; TN was exceeded in outlet 2; and TP exceeds the limit concentration more than twice in the outflows from both outlets.
- The concentration of petroleum hydrocarbons expressed as HOI for both outlets was exceeded compared to the limit value. The indicator was nearly three times higher for outlet 2 from a more heavily urbanized residential–commercial area with a developed transportation network.
- Zn concentrations were exceeded compared to the limit for both outlets.
- The concentrations of the other heavy metals tested (Cu and Hg) in outlet 1 were below the minimum quantification level; in outlet 2, the concentrations were exceeded compared to the limits.
- The problems, in turn, are the high concentrations of PAHs, which for outlet 1 and outlet 2 were higher than the limit value and, comparing the concentrations for both outlets, it can be seen that they are nearly four times higher for outlet 2 from a more heavily urbanized residential–commercial area with a developed transportation network.

For the rest of the studied indicators, no limits are defined, their comparative evaluation was performed (Table 10), and the following relationships were obtained:

- TSS concentrations were more than 3.5 times higher for outlet 2 from a more heavily urbanized residential–commercial area with a developed transportation network;
- COD was also about three times higher for outlet 2;
- Kjeldahl nitrogen concentration TKN and N–NO_2_ concentration were similar for both outlets.

### 3.5. Estimation of the Impact of Polluted Runoff on River Quality

**Figure 9:**
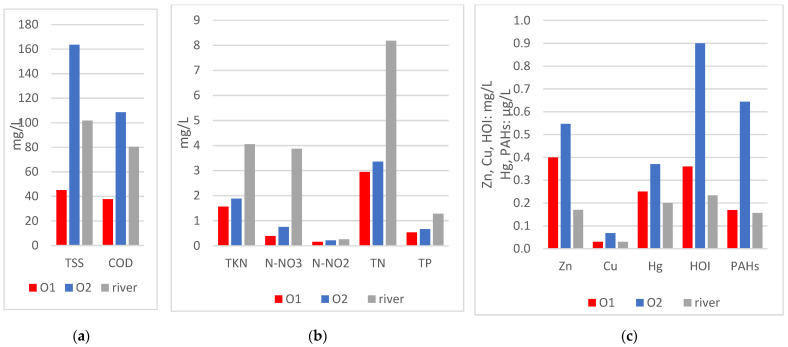
Comparison of average concentrations of pollutant indicators in stormwater from outlets O1 and O2 and in the river: (**a**) concentrations of suspended solids and COD; (**b**) concentrations of biogenic compounds; (**c**) concentrations of heavy metals, HOI, and PAHs.

Based on the results of the study, an estimate was made of the impact of polluted runoff on water quality in the Sudół River. Figure 9 presents the average concentrations of pollutant indicators calculated from the measurements (Table 7, Table 8 and Table 9). The highest pollutant concentrations for almost all pollutant indicators tested (except biogenic compounds) are found in outlet 2, which discharges stormwater from a highly urbanized catchment area including residential, commercial, and high-traffic roads (Figure 9a,c). Biogenic pollutants have the highest average concentrations in the river’s waters (Figure 9b). Pollutant concentrations from outlet 1 (residential area) are higher than in the river for HOI, Zn, Hg, and PAHs, and lower than in the river for indicators TSS, COD, and TN.

**Figure 10:**
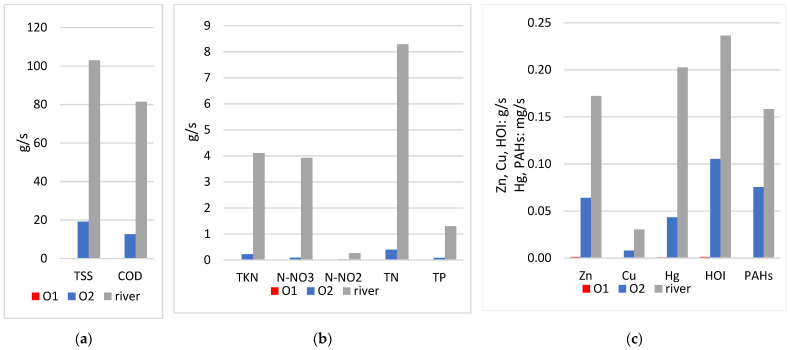
Average loads of pollutant indicators discharged during rainfall with *p* = 20% in stormwater from outlets O1 and O2 and in the river: (**a**) loads of TSS and COD; (**b**) loads of biogenic compounds; (**c**) loads of heavy metals, HOI, and PAHs.

Based on the average concentrations in the sewage basins and in the river shown above, and based on the results of hydrological modeling (Table 6), the average loads (according to Formula (7)) that run off in surface runoff from the residential area and the service and transportation area were calculated, as well as the pollutant loads in the river. The results are illustrated in the graphs below (Figure 10).

Estimated pollution load calculations demonstrate:

- The amount of load depends not only on the concentration, but also on the size of the outflow, so the contribution of outlet 1, which discharges stormwater from a settlement with a small area, is insignificant (less than 1% for all pollution indicators).
- The total loads discharged from the two studied outlets of the rainfall sewer system may account for 2% to as much as 48% of the loads present in the waters of the Sudół River below the mouth of these outlets.
- The lowest contribution to river loads from outlets 1 and 2 is estimated in biogenic compounds, from 2–10%, Figure 10b, as the river is more polluted than the discharged stormwater, as also shown in the concentration graph in Figure 9b.
- The share of loads from outlets 1 and 2 in the amount of TSS and COD is 19% and 16%, respectively (Figure 10a).
- The highest share of pollutant loads discharged in stormwater from outlets 1 and 2 in the river loadings is for PAH, i.e., 48% and 45% share for HOI, respectively, and for heavy metals: 22% for Hg, 38% for Zn, and 27% for Cu (Figure 10c). The results show how significant the impact can be of untreated storm drain runoff from sealed heavily polluted areas such as roads, parking lots, and other impervious surfaces.

### 3.6. Environmental Risk Assessment

The RQ values calculated for water samples from surface runoff and the river allowed us to determine the probability of negative effects in the ecosystem due to exposure to a given pollutant. PNEC values taken from the literature are included in Table 4.

Table 11 shows the estimated RQ values for a given compound. When estimating environmental risk values, it is assumed that if the concentrations of MEC are higher than the PNEC, they may cause hazardous effects on aquatic organisms. 

In order to best determine the environmental risk, three scenarios were developed that represent the possibility of negative effects for the optimistic, pessimistic, and most likely outlook. For the optimistic scenario, the RQ is calculated for an MEC equal to the lowest determined value of the parameter in question; for the pessimistic scenario, the highest value is taken as the MEC, while for the most likely, and therefore realistic, the MEC is the average. If the MEC is below the MQL, then no RQ was calculated for the minimum value; for the maximum value, MEC equal to the MQL was taken, while the average value of the measurement result was set at half the value of the given limit of determination [99]. 

The optimistic scenario, i.e., assuming the least possible environmental impact, yields convergent results for outlets 1 and 2. Only Zn is estimated to slightly cause adverse effects in the ecosystem, and in turn, N–NO_3_ shows an RQ almost 6 times higher for outlet O2 than O1, showing significant and low potential for adverse effects in the ecosystem, respectively. For the river, the RQ of N–NO_3_ is the highest, at more than twice the lower limit at which adverse effects in the ecosystem should be expected. 

The pessimistic scenario has the most adverse effects of the analyzed micropollutants on aquatic organisms. For all compounds, except HOI, ecological risk occurs because RQ > 1. For the primary pollutants (except nitrate nitrogen), the RQ for outlet O2 is 1.4 to 3.7 times higher than for outlet O1 and the river. N–NO3 shows the highest RQ for the river, which is as much as five times the limit above which ecosystem impacts are most likely to occur. 

Calculations for the realistic scenario, which is a good approximation of the real conditions in the study catchments, show that the aquatic ecosystem is at risk. The risk factor for primary pollutants ranged from 1.5 for TSS in outlet O1 to as high as 387.3 for N–NO_3_ in the river. For heavy metals, the RQ took values ranging from a few to dozens, with the lowest values obtained for Hg and the highest for Cu. 

## 4. Discussion

**Figure 11:**
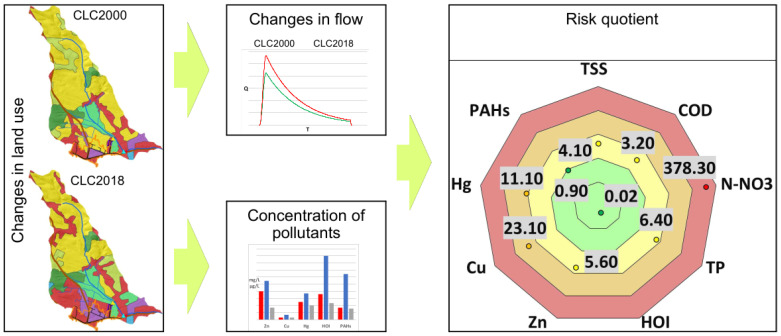
Graphical illustration of the conducted analyses.

Figure 11 shows the analyses carried out for the Sudół River catchment case study, which is subject to intensive changes in land use in Krakow.

The analysis of CLC 2000 and 2018 shows an increase in sealed areas (Figure 7 and Figure 8 and Table 5), which causes an increase in flow (Table 6). In the section closing the catchment for the analyzed design precipitation of 19.38 mm (with *p* = 20% and duration of 15 min), an increase in flow of 2.62% was estimated, but in selected areas it may cause a larger increase in runoff, e.g., analyzed outlet 2 may increase by 16.22%. Similar analyses were performed by, among others, Ociepa and Suligowski for the urbanized catchment area in Kielce, Poland [11,100], Sjöman and Gill for a catchment area in Sweden [67], and Li et al. for the city of Shenyang in China [66]. However, the results are difficult to compare due to the individual nature of each location (land use and land cover, soils, climate, etc.). 

The main objective of this work was to investigate the qualitative aspects of stormwater. Stormwater discharged from areas of different land use have different quality parameters. Measurement campaigns were undertaken to determine the concentrations of 12 key parameters: (1) the quality of stormwater in the outflows from the drainage system from two urban areas of different sizes and different land uses, and (2) the water quality of the Sudół River, to which the stormwater is discharged. The results of 10 measurements of the quality of stormwater are presented in Table 8 and Table 9. The obtained results of the study were also referenced in a review study by De Buyck et al. in 2021 [101], which reviewed 39 publications from 1999–2019, based on which, among others, the average and maximum concentrations of pollutants in stormwater were calculated. A comparison of the obtained values of the average and maximum concentrations of the pollutants studied in the present study and the calculations made by De Buyck et al. is presented in Table 12. In order to relate the obtained results of the study to previous Polish studies, a comparison was made with the results of Strzebońska et al. [102], who conducted a study of the quality of roof runoff in Krakow, and studies of the quality of stormwater in cities by Poznań [103], Częstochowa [104], and Kielce [105].

The calculated mean and maximum concentrations from all measurements (outlet 1 and outlet 2) show higher values for all tested biogenic compounds; additionally, the determined mean concentration for COD is higher than in the work of De Buyck et al. 

In the study [102], 31 pollutant indicators were determined, including N–NO_3_, Cu, and Zn indicators in common with the present work. Demonstrated concentrations in roof runoff, which should be of better quality than the runoff studied in our work covering runoff from rooftops, roads, and parking areas, were lower for N–NO_3_ and Cu indicators, while Zn concentrations were higher. 

The determined concentrations in outlets 1 and 2 were also compared to other studies on stormwater quality in Polish cities, presented in Table 12:

- The study in Poznań [103] includes 8 parameters in common with the present work. The results of stormwater quality in Krakow were worse in terms of average concentrations for indicators TSS, TKN, N–NO_2_, TP, and Cu; however, significant differences are found for N–NO_2_: more than 18 times higher mean and max concentration; TP: more than 2.7 times higher mean concentration and 4.8 times maximum concentration; and Cu: more than 2 times higher mean concentration, but the recorded maximum concentration is lower by half.
- In the study in Częstochowa [104], three parameters common to this study were taken into account: TSS, COD, and Cu. The results of the quality of stormwater in Krakow were worse in terms of mean Cu concentration (10 times higher), COD with similar mean concentration, and TSS with a two-times lower concentration.
- In the study in Kielce [105], four parameters common to this study were taken into account: TSS, Zn, Cu, and Hg. The results of stormwater quality in Krakow were better in terms of average concentrations for TSS (just below the lower limit of the range of mean concentrations), Cu (about 2 times lower than the lower limit of the range of mean concentrations), Hg (over 1000 times the lower limit of the range of mean concentrations), and Zn (at the upper limit of the range of average concentrations).

The analyses carried out and the concentrations obtained prove that land use has an impact on the quality of stormwater and, as a result, on the quality of surface water. Concentrations from two drainage outlets were examined: a small residential area (outlet 1) and a residential and commercial area with a developed transportation network with heavy traffic (outlet 2). The comparison made in Table 10 and Figure 9 shows that the more intensive development (outlet 2), which includes, e.g., commercial areas and high-traffic roads, results in average concentrations higher than in low-density residential areas (outlet 1). In particular, this applies to such pollutants as TSS, COD, HOI, Cu, and PAHs; their average concentrations were more than two times higher in outlet 2 than in outlet 1. Similar conclusions were obtained, e.g., in a study for different types of land use in Singapore [106]: concentration in stormwater from residential area is lower than from areas such as business districts, industry, and residential roads in term of parameters TSS, Zn, and Cu. A similar study was also performed by Wang et al. [13] showing that the average concentrations of TSS, COD, Zn, and Cu in runoff of rainwater in Chongqing (China) from urban traffic roads are much higher than from residential roads, commercial areas, and roof runoff. Paule et al. [15] studied the relationship between land use change and stormwater runoff quality in Yongin, South Korea. A correlation has been shown between the increase in concentrations of TSS, COD, TN, and TP and the increase in commercial, parking lot, residential, and road areas.

Threats to aquatic ecosystems were investigated through environmental risk assessment for stormwater discharged through outlets 1 and 2 and the Sudół River. The magnitude of the RQ for COD, TP, and N–NO_3_ was calculated, taking into account the limits for waters in which freshwater fish can live. Due to the high concentrations of N–NO_3_, it is reasonable to believe that this compound could cause negative effects among fish. In the river, the RQ is more than three times the values for which such an impact should be expected. Despite the fact that phosphorus and nitrogen are essential nutrients, their excess in the waters leads to eutrophication. Algal blooms limit the development of shallow-water vegetation and produce poisonous substances that are a threat to animal organisms and human health and life [107]. A significant amount of suspended matter in the water is not toxic in itself, but the threat is posed by various substances sorbing on it that are dangerous to the aquatic ecosystem [108]. In the studies conducted, a positive correlation between RQ for suspended solids and heavy metals is noticeable. Cu compounds can cause significant risks to the aquatic environment. They are considered harmful to aquatic ecosystems, and crustaceans are considered the most sensitive organisms [95]. Fish, on the other hand, exhibit a wide range of toxicity values, but their ability to reproduce and grow can be impaired when chronically exposed to Cu [109]. Zn shows toxicity to aquatic organisms, especially plankton [110]. According to Gebar et al. [91], a negative effect occurs in half of the arthropod population studied at an RQ of 7.3 calculated according to the PNEC adopted by the authors. In the calculations carried out for the realistic scenario, this value was exceeded at least twice, which clearly suggests that a negative effect of exposure of living organisms to this element is very likely to occur. Another highly toxic metal is Hg, and its presence in surface waters poses a threat to living organisms. Its compounds can accumulate in mollusks, fish, and successively further up the food chain to humans [111]. Hg concentrations at ng/L levels cause toxicity in Daphnia [112], so of the three freshwater locations studied, these organisms are most vulnerable in river waters. In contrast, a study by Zhang et al. shows that fish have a higher tolerance to Hg than do phytoplankton and invertebrates [113]. In addition to heavy metals, PAHs are well-known contaminants due to their strong carcinogenic and mutagenic properties [97]. These compounds, despite their low water solubility and hydrophobicity, have been found in surface waters. The results obtained for the realistic scenario correlate with the literature data. The RQ for four select PAHs in Yellow River waters in China is <1 [114], while in Brazil it is up to 4 [97].

The conducted research proves that urbanization and the accompanying changes in land use have led to changes in hydrology and increased pollution of surface waters, and this may pose a threat to aquatic ecosystems in the Sudół River catchment. For this reason, it is important to introduce stormwater management rules to stop such negative trends and reduce threats. There are many studies that show the beneficial effect of the use of stormwater control measures (SCMs) on reducing pollution and surface runoff. Pennino et al. [115] indicate that the use of stormwater green infrastructure brings a significant reduction in flash hydrology and pollution concentration. SCMs reduce the concentration of phosphorus [116,117], and they can limit, delay, or stabilize the supply of nitrogen [115,118,119,120]; in the case of suspension, no influence on their reduction is shown [115,116], but of course it depends on the type of SCMs, their location, and the scale of the solutions used [115,118,119]. A study by Walsh et al. [116] showed that extensive use of dispersed SCMs can reverse the negative effects of urbanization and improve stream water quality. Therefore, it seems advisable to introduce administrative recommendations (or even an obligation) to apply stormwater control measures for all new investments, as well as to strengthen their implementation through economic instruments, such as rainwater charges and investment co-financing. Economic incentives can also induce owners of already built-up real estate to change their stormwater management. As we have shown in our previous work [121,122,123], the existing economic instruments in Poland need to be changed in order to effectively encourage property owners to invest in sustainable rainwater management.

## 5. Conclusions

An assessment of the impact of land use changes and stormwater management in selected developed areas (a small residential area and a larger residential–commercial area with an intensive traffic network) on the surface water quality of the Sudół River was conducted, with the following findings:

- The changes in land use from 2000 to 2018 were estimated at the scale of the entire catchment and their impact on the change in sealing and changes in hydrology, showing that progressive urbanization has resulted in the conversion of land used for agriculture into residential land (an increase of more than 96%) and industrial and commercial land (an increase of 113%), resulting in an increase in the degree of sealing (the CN curve at the scale of the entire catchment changed from 77.72 to 78.28), which is reflected in an increase in surface runoff and flows in the river (hydrological modeling for precipitation with *p* = 20% shows a 2.6% increase in flow in the estuary section of the catchment).
- Changes in development lead to changes in hydrology: a clear impact was found from the analyses in 1 of the 2 areas studied: the residential–commercial area, where 31.76 ha of land changed its use in the period 2000–2018 (which accounts for 40% of the area), resulting in changes in the CN curve from a value of 85.47 to 88.05 and a 16% increase in outflow from the stormwater drainage system for *p* = 20% rainfall.
- We conducted a study of the quality of stormwater discharges from the analyzed 2 areas to show significant pollution, in particular, in terms of such pollutants as TSS (average concentration in outlet 1: 45 mg/L, in outlet 2: 164 mg/L), petroleum hydrocarbons (HOI in O1: 0.36 mg/L; in O2: 0.9 mg/L), PAHs (in O1: 0.1689 µg/L; in O2: 0.6438 µg/L), and heavy metals (Cu in O1: 0.03 mg/L; in O2: 0.0685 mg/L, Zn in O1: 0.4 mg/L; in O2: 0.547 mg/L, and Hg in O1: 0.0003 mg/L; in O2: 0.0004 mg/L). Concentrations of these pollutants in particular from outlet 2 from a residential–commercial area with a heavy traffic transportation network were 2 times (TSS, Cu, and Hg), 3 times (Zn), and even 4 times (HOI and PAHs) higher than in the waters of the Sudół River.
- Estimated pollutant loads contributed by stormwater may account for a significant share of the loads observed in the river in the 130 m cross-section downstream of outlet 2. Calculations conducted for precipitation *p* = 20% and average concentrations show that outlet 2, draining from a highly urbanized, sealed catchment, may account for more than 40% of the load of petroleum hydrocarbons and PAHs, as well as 21–37% of the load of heavy metals analyzed.
- Environmental risk assessment of surface runoff and waters of the Sudół catchment shows the highest risks for N–NO_3_, with the highest risk found in the river waters. High risks are also shown for heavy metals, the highest for Cu concentrations in stormwater discharged by outlet 2; for this outlet, a significant level of risk is found for Zn. For waters from outlet 1, a significant level of risk is found for Zn and Cu.

The existing approach to stormwater management in the form of its discharge directly into the waters of the Sudół River and drainage ditches without treatment may be responsible for the exceedances of permissible concentrations in the river in terms of the indicators TN, TP, Zn, Cu, Hg, petroleum hydrocarbons (HOI), and polycyclic aromatic hydrocarbons (PAHs), as the recorded concentrations of these indicators in particular in outlet 2 exceed the concentration limits allowed for Class II surface water.

Such problems are likely to occur in 1/3 of the Sudół catchment area—this percentage is currently made up of residential neighborhoods and industrial and commercial areas. If the catchment area is subjected to further continuous development, this may contribute to the persistence of poor physical and chemical status or even its deterioration, and thus contribute to the threat of not achieving good water status in the Sudół catchment area.

Development is inevitable, but it is necessary to strive for stormwater management that will limit surface runoff and reduce its pollution. The use of green infrastructure can reduce stormwater pollution [124,125], as studies show that it is possible to apply solutions that can reduce both biogenic pollution [20,120,126] and substances such as heavy metals [127,128] and PAHs [35,129].

## Figures and Tables

**Table 1:** Summary of the permissible values characterizing the good status (class II) of surface water quality for JCWP Prądnik.

Parameter	Unit	Limit Value for Good Status (Class II)
Nitrate nitrogen	mg N–NO_3_/L	<2.0
Total nitrogen	mg N/L	<3.0
Total phosphorus	mg P/L	<0.25
Zinc	mg Zn/L	<0.1
Copper	mg Cu/L	<0.01
Petroleum hydrocarbons—hydrocarbon oil index (HOI)	mg/L	<0.2
Mercury	μg/L	<0.07 (maximum)
Polycyclic aromatic hydrocarbons (PAHs)—benzo(a)pyrene	μg/L	<0.00017 (mean) <0.27 (maximum)

**Table 2:** Selected stormwater priority pollutants (indicator parameters) [36].

No.	Type	Name
1	Basic parameters	pH, Biochemical oxygen demand BOD, Chemical oxygen demand COD, Suspended solids, Nitrogen, Phosphorus
2	Metals	Zinc Zn, Cadmium Cd, Chromium as Chromate Cr(IV), Copper Cu, Nickel Ni, Lead Pb, Platinum Pt
3	Polycyclic aromatic hydrocarbons (PAHs)	Benzo[a]pyrene, Naphthalene, Pyrene
4	Herbicides	Terbutylazine, Pendimethalin, Phenmedipham, Glyphosate
5	Miscellaneous	Nonylphenol ethoxylates and degradation products, Pentachlorophenol, Di(2-ethylhexyl) phthalate, Polychlorinated biphenyl 28, Methyl tert-butyl ether

**Table 3:** CN parameter values for a land use typical of an urban catchment area [69].

Cover Description		Curve Numbers for Hydrologic Soil Group			
Cover Type and Hydrologic Condition	Average Percent Impervious Area	A	B	C	D
Open space (lawns, parks, cemeteries, etc.):					
Poor condition (grass cover <50%)		68	79	86	89
Poor condition (grass cover 50% to 75%)		49	69	49	84
Poor condition (grass cover >75%)		39	61	74	80
Impervious areas:					
Paved parking lots, roofs, driveways, etc. (excluding right-of-way)		98	98	98	98
Street and roads:		98	98	98	98
Paved; curbs and storm sewers (excluding right-of-way)					
Paved; open ditches (including right-of-way)		83	89	92	93
Gravel (including right-of-way)		76	85	89	91
Dirt (including right-of-way)		72	82	87	89
Urban district:					
Commercial and business	85	89	92	94	95
Industrial	72	81	88	91	93
Residential district by average lot size:					
1/8 acre or less (506 m^2^ or less)	65	77	85	90	92
1/4 acre (1012 m^2^)	38	61	75	83	87
1/3 acre (1349 m^2^)	30	57	72	81	86
1/2 acre (2023 m^2^)	25	54	70	80	85
1 acre (4047 m^2^)	20	51	68	79	84
2 acres (8094 m^2^)	12	46	65	77	82

**Table 4:** PNEC values for selected surface water pollutants.

Parameter	PNEC (mg/L)	Source
TSS	25	[92]
COD	25	[93]
TN	0.01	[92]
TP	0.2	[92]
HOI	10	[94]
Zn	0.0302	[95]
Cu	0.0013	[95]
Hg	0.00018	[96]
PAHs	0.00017	[97]

**Table 5:** Summary of parameters for individual calculation sections.

Cross-Section on the Sudół River	Area (km^2^)	CN CLC 2000	CN CLC 2018
Residential development: outlet 1	0.042	83.74	83.74
Residential and commercial area: outlet 2	1.203	85.47	88.05
Sudół: cross-section A	17.960	76.97	77.58
Sudół: whole catchment	19.090	77.72	78.28

**Table 6:** Summary of the results of flow calculations for the cross-sections of the Sudół River catchment area.

Cross-Section on the Sudół River	CLC 2000		CLC 2018		Flow Increment in Years 2000–2018
	Q_max_	Outflow Volume	Q_max_	Outflow Volume	
	m^3^/s	m^3^	m^3^/s	m^3^	
Residential development: outlet 1	0.003	230.50	0.003	230.60	0.05%
Residential and commercial area: outlet 2	0.101	7225.44	0.117	8397.12	16.22%
Sudół: cross-section A	0.985	70,767.40	1.013	72,751.35	2.80%
Sudół: whole catchment	1.084	77,812.70	1.112	79,848.22	2.62%

**Table 7:** Results of studies of water quality of the Sudół River: measured, minimum, maximum, and average concentrations of selected indicators of pollution.

Sample Code	Month of Sampling	TSS	COD	TKN	N–NO_3_	N–NO_2_	TN	TP	HOI	Zn	Cu	Hg	PAHs
		mg/L	mg/L	mg/L	mg/L	mg/L	mg/L	mg/L	mg/L	mg/L	mg/L	mg/L	µg/L
737	Mar 2022	180	165.0	3.300	2.180	0.121	5.60	1.07	0.50	0.33	<MQL	<MQL	0.350
1110	May 2022	74	49.2	7.930	5.020	0.545	13.50	1.99	<MQL	0.09	<MQL	<MQL	0.028
1917	Sep 2022	51	26.9	0.919	4.420	0.108	5.45	0.78	<MQL	0.09	<MQL	<MQL	0.091
Min		51	26.9	0.919	2.180	0.108	5.45	0.78	<MQL	0.09	<MQL	<MQL	0.028
Max		180	165.0	7.930	5.020	0.545	13.50	1.99	0.50	0.33	<MQL	<MQL	0.350
Mean *		102	80.4	4.050	3.873	0.258	8.183	1.28	0.233	0.17	0.03	0.0002	0.1563

<MQL: below the minimum quantification level (MQL); *: <MQL were substituted with MQL/2 in calculations of mean values [99].

**Table 8:** Results of stormwater quality examination from outlet 1 (residential area): measured, minimum, maximum, and average concentrations of selected pollutant indicators.

Sample Code	Month of Sampling	TSS	COD	TKN	N–NO_3_	N–NO_2_	TN	TP	HOI	Zn	Cu	Hg	PAHs
		mg/L	mg/L	mg/L	mg/L	mg/L	mg/L	mg/L	mg/L	mg/L	mg/L	mg/L	µg/L
293	Feb 2020	6	9.0	0.409	nt	nt	nt	0.063	0.70	0.39	<MQL	<MQL	nt
309	Feb 2021	120	82.8	5.620	0.444	0.066	6.130	1.200	0.60	0.78	<MQL	<MQL	0.310
388	Feb 2022	29	15.7	1.780	0.086	<MQL	1.780	1.780	<MQL	0.28	<MQL	<MQL	0.160
736	Mar 2022	99	131.0	2.300	0.566	0.056	2.920	0.250	<MQL	0.71	<MQL	<MQL	0.240
1109	May 2022	20	43.0	3.950	0.430	0.055	4.440	0.418	<MQL	0.55	<MQL	<MQL	0.021
1926	Aug 2019	10	8.2	0.385	nt	nt	nt	0.129	<MQL	0.11	<MQL	<MQL	nt
1916	Sep 2022	95	43.2	0.825	0.734	0.056	1.620	0.780	0.40	0.36	<MQL	<MQL	0.048
2134	Sep 2019	8	6.3	0.220	nt	nt	nt	0.278	0.40	0.31	<MQL	<MQL	0.160
2074	Nov 2020	48	26.1	<MQL	0.046	0.712	0.758	0.239	0.30	0.27	<MQL	<MQL	0.360
2551	Nov 2019	16	11.3	<MQL	nt	nt	nt	0.208	0.80	0.24	<MQL	<MQL	0.052
Min		6	6.3	0.046	0.046	<MQL	0.758	0.063	<MQL	0.11	<MQL	<MQL	0.021
Max		120	131.0	5.620	0.734	0.712	6.130	1.780	0.80	0.78	<MQL	<MQL	0.360
Mean *		45	37.7	1.558	0.384	0.158	2.941	0.535	0.36	0.40	0.03	0.0003	0.1689

nt: not tested; <MQL: below the minimum quantification level (MQL); *: <MQL were substituted with MQL/2 in calculations of mean values [99].

**Table 9:** Results of stormwater quality examination from outlet 2 (residential–commercial area): measured, minimum, maximum, and average concentrations of selected pollutant indicators.

Sample Code	Month of Sampling	TSS	COD	TKN	N–NO_3_	N–NO_2_	TN	TP	HOI	Zn	Cu	Hg	PAH
		mg/L	mg/L	mg/L	mg/L	mg/L	mg/L	mg/L	mg/L	mg/L	mg/L	mg/L	µg/L
292	Feb 2020	58	34.0	0.731	nt	nt	nt	0.214	0.8	0.24	<MQL	0.0003	nt
308	Feb 2021	320	252.0	2.230	0.473	0.075	2.78	0.852	2.1	1.19	0.180	<MQL	1.100
387	Feb 2022	440	214.0	3.390	0.258	0.029	3.68	2.720	1.2	0.94	0.115	<MQL	1.300
735	Mar 2022	440	308.0	3.900	0.946	0.198	5.04	1.090	2.1	1.06	0.150	<MQL	0.880
1108	May 2022	31	113.0	3.550	1.380	0.208	5.14	0.505	0.2	0.65	<MQL	<MQL	0.032
1925	Aug 2019	18	7.2	2.420	nt	nt	nt	0.390	<MQL	0.12	<MQL	<MQL	nt
1915	Sep 2022	16	12.1	0.852	1.020	0.041	1.91	0.185	<MQL	0.23	<MQL	<MQL	0.048
2133	Sep 2019	35	18.6	1.120	nt	nt	nt	0.146	0.8	0.41	<MQL	<MQL	0.840
2073	Nov 2020	180	74.4	0.426	0.436	0.711	1.570	0.434	0.6	0.37	0.060	0.0007	0.760
2550	Nov 2019	98	51.6	0.169	nt	nt	nt	0.071	1.0	0.26	<MQL	0.0007	0.190
Min		16	7.2	0.169	0.258	0.029	1.57	0.071	<MQL	0.12	<MQL	<MQL	0.0032
Max		440	308.0	3.900	1.380	0.711	5.14	2.720	2.1	1.19	0.180	0.0007	1.300
Mean *		164	108.5	1.879	0.752	0.210	3.353	0.661	1.1	0.547	0.1263	0.0006	0.644

nt: not tested; <MQL: below the minimum quantification level; *: <MQL were substituted with MQL/2 in calculations of mean values [99].

**Table 10:** Comparison of the determined average concentrations of pollutants analyzed in the outflow from the residential area and the residential–commercial area.

Parameter	Unit	Concentration in Outlet O1	Concentration in Outlet O2	O1/O2 Concentration Ratio
TSS	mg/L	45	164	3.6
COD	mg/L	37.7	108.5	2.9
TKN	mg/L	1.558	1.879	1.2
N–NO_3_	mg/L	0.384	0.752	2.0
N–NO_2_	mg/L	0.158	0.210	1.3
TN	mg/L	2.941	3.353	1.1
TP	mg/L	0.535	0.661	1.2
HOI	mg/L	0.36	0.90	2.5
Zn	mg/L	0.400	0.547	1.4
Cu	mg/L	0.0300	0.0685	2.3
Hg	mg/L	0.0003	0.0004	1.5
PAHs	µg/L	0.1689	0.6438	3.8

**Table 11:** Calculated risk quotient values for three sampling locations for stormwater and surface water allowing for assessment of potential ecological risk.

Parameter	RQ for Outlet 1			RQ for Outlet 2			RQ for River		
	Scenario								
	Realistic	Pessimistic	Optimistic	Realistic	Pessimistic	Optimistic	Realistic	Pessimistic	Optimistic
TSS	1.8	4.8	0.2	6.5	17.6	0.6	4.1	7.2	3.0
COD	1.5	5.2	0.3	4.3	12.3	0.3	3.2	6.6	2.0
N–NO_3_	38.4	73.4	4.6	75.2	138.0	25.8	387.3	502.0	218.0
TP	2.7	8.9	0.3	3.3	13.6	0.4	6.4	10.0	5.4
HOI	0.04	0.1	<MQL	0.1	0.2	<MQL	0.02	0.1	<MQL
Zn	13.2	25.8	3.6	18.1	39.4	4.0	5.6	10.9	3.0
Cu	23.1	46.2	<MQL	97.2	138.5	<MQL	23.1	<MQL	<MQL
Hg	8.9	22.2	<MQL	3.3	3.9	<MQL	11.1	<MQL	<MQL
PAHs	1.0	2.1	0.1	3.8	7.6	0.2	0.9	2.1	0.2
Adopted evaluation scale:									
no environmental risk	low potential for adverse effects			considerable potential for adverse effects			adverse effects are expected		
RQ < 1	1 ≤ RQ < 10			10 ≤ RQ < 100			RQ ≥ 100		

**Table 12:** Comparison of the determined maximum and average concentrations of pollutants in the analyzed outflows and literature.

Parameter	Unit	Outlets 1 and 2		Review of Global Research [101]		Polish Research [102,103,104,105]	
		Mean	Max	Mean	Max	Mean	Max
TSS	mg/L	104.350	440	124.937	1130	16.85–55.31 [103] 195.59 [104] 106.00–5514.00 [105]	736 [103] 410 [104] 7432 [105]
COD	mg/L	73.077	308	68.93	360.2	75.348 [104]	129 [104]
TKN	mg/L	1.718	5.620	1.049	2.800	1.240–1.701 [103]	5.540 [103]
N–NO_3_	mg/L	0.568	1.380	0.010	0.010	0.440–0.900 [103]	0.091 [102] 4.110 [103]
N–NO_2_	mg/L	0.184	0.712	1.556	2.170	0.010 [103]	0.040 [103]
TN	mg/L	3.147	6.130	2.561	8.744	2.690–3.580 [103]	14.600 [103]
TP	mg/L	0.598	2.720	0.376	1.757	0.120–0.220 [103]	0.570 [103]
HOI	mg/L	0.630	2.100				
Zn	mg/L	0.474	1.190	0.184	1.979	0.029–0.697 [103] 0.158–0.473 [105]	0.091 [102] 7.820 [103] 0.858 [105]
Cu	mg/L	0.049	0.180	0.036	0.645	0.006–0.024 [103] 0.005 [104] 0.089–0.195 [105]	0.091 [102] 0.326 [103] 0.008 [104] 0.320 [105]
Hg	mg/L	0.0003	0.0007	0.008	0.100	0.304–0.992 [105]	1.405 [105]
PAHs	µg/L	0.406	1.300	1.828	9.570		

## Data Availability

Not applicable.

## Abbreviations

COD—Chemical oxygen demand, HOI—Hydrocarbon oil index, N–NO_2_—Nitrite nitrogen, N–NO_3_—Nitrate nitrogen, PAHs—Polycyclic aromatic hydrocarbons, P–PO_4_—Phosphate phosphorus, TKN—Kjeldahl nitrogen, TN—Total nitrogen, TP—Total phosphorus, TSS—Total suspended solids.
